# Tailored Levofloxacin Incorporated Extracellular Matrix Nanoparticles for Pulmonary Infections

**DOI:** 10.3390/ijms26157453

**Published:** 2025-08-01

**Authors:** Raahi Patel, Ignacio Moyano, Masahiro Sakagami, Jason D. Kang, Phillip B. Hylemon, Judith A. Voynow, Rebecca L. Heise

**Affiliations:** 1 Department of Biomedical Engineering, Virginia Commonwealth University, Richmond, VA 23219, USA; patelr38@vcu.edu (R.P.); moyanoig@vcu.edu (I.M.); 2 Department of Pharmaceutics, Virginia Commonwealth University, Richmond, VA 23219, USA; msakagam@vcu.edu; 3 Stravitz-Sanyal Institute for Liver Disease & Metabolic Health, School of Medicine, Virginia Commonwealth University, Richmond, VA 23219, USAphillip.hylemon@vcuhealth.org (P.B.H.); 4 Department of Microbiology and Immunology, School of Medicine, Virginia Commonwealth University, Richmond, VA 23219, USA; 5 Division of Pediatric Pulmonology, Children’s Hospital of Richmond at Virginia Commonwealth University, Richmond, VA 23298, USA; judith.voynow@vcuhealth.org

**Keywords:** nanoparticles, pulmonary delivery, mucus barrier, levofloxacin, extracellular matrix, cystic fibrosis

## Abstract

Cystic fibrosis produces viscous mucus in the lung that increases bacterial invasion, causing persistent infections and subsequent inflammation. Pseudomonas aeruginosa and Staphylococcus aureus are two of the most common infections in cystic fibrosis patients that are resistant to antibiotics. One antibiotic approved to treat these infections is levofloxacin (LVX), which functions to inhibit bacterial replication but can be further developed into tailorable particles. Nanoparticles are an emerging inhaled therapy due to enhanced targeting and delivery. The extracellular matrix (ECM) has been shown to possess pro-regenerative and non-toxic properties in vitro, making it a promising delivery agent. The combination of LVX and ECM formed into nanoparticles may overcome barriers to lung delivery to effectively treat cystic fibrosis bacterial infections. Our goal is to advance CF care by providing a combined treatment option that has the potential to address both bacterial infections and lung damage. Two hybrid formulations of a 10:1 and 1:1 ratio of LVX to ECM have shown neutral surface charges and an average size of ~525 nm and ~300 nm, respectively. The neutral charge and size of the particles may suggest their ability to attract toward and penetrate through the mucus barrier in order to target the bacteria. The NPs have also been shown to slow the drug dissolution, are non-toxic to human airway epithelial cells, and are effective in inhibiting *Pseudomonas aeruginosa* and *Staphylococcus aureus*. LVX-ECM NPs may be an effective treatment for pulmonary CF bacterial treatments.

## 1. Introduction

Cystic fibrosis (CF) is a genetic disorder caused by a mutation in the cystic fibrosis transmembrane conductance regulator (CFTR) gene and roughly affects over 40,000 people in the United States [1]. The mutated protein disrupts chloride and bicarbonate movement across the membrane of cells. Specifically, it is unable to move chloride ions outside of the cells, which fails to attract water to the apical surface, causing the mucus of multiple organs to become thick and sticky [1]. In the lungs, the thickened mucus causes blockages that contribute to lung damage while also increasing the opportunity for bacterial invasion which can result in persistent infections, inflammation, and respiratory failure [2]. Two of the most common bacterial strains are Pseudomonas aeruginosa and Staphylococcus aureus. These bacterial infections severely impair lung function and contribute to chronic infection resistant to antibiotic therapies.

The extracellular matrix (ECM) is a matrix scaffold of several proteins that allows for the migration, proliferation, and differentiation of cells, providing a stable microenvironment for cells and tissues in the body to maintain homeostasis [3].The ECM in a CF environment is heavily dysregulated due to matrix structure and composition changes via cell, tissue, and organ dysfunction [4]. Matrix breakdown and thickening of the layers of the ECM are speculative root causes of airway remodeling, or the thickening and widening of CF lung airways that cause further impairment to normal function [5].

CF treatment includes a wide range of therapies to control symptoms, but a critical target is treating infections with antibiotics through intravenous, oral, or inhalation pathways. Inhalation via aerosolized particles is the preferred method, where nanoparticles (NPs) are an emerging inhaled therapeutic due to their capacity for more optimal drug deposition into the lower lungs. NPs may also be tailorable depending on disease state, target locations, and treatment options, making them a highly versatile therapeutic. One of the biggest barriers of inhaled particle deposition is mucus, governed by size and interaction filtering [6]. CF mucus mesh size is decreased (100 to 400 nm) due to high amounts of DNA entanglement, meaning particles larger than the pore size become rapidly immobilized [7]. CF mucus also contains anionic glycosylated regions and periodic hydrophobic domains within mucin fibers, hindering the diffusivity of charged particles [7,8,9].Therefore, creating NPs less than 400 nm in diameter and neutrally charged (−10 mV and +10 mV) through electrospray deposition may attract and penetrate through to target infections.

To tackle infections, levofloxacin (LVX) was chosen as the first component of the hybrid NPs, as it inhibits bacterial replication [10]. Levaquin^®^ tablets, oral solution, injection, and IV pathways are FDA-approved for treating pneumonia and several bacterial infections [11]. Additionally, a nebulized LVX solution (Quinsair^®^) has been approved for CF patients in the US, European Union, and Canada to treat long-term *P. aeruginosa* infections [12]. However, non-inhalation pathways and nebulized solutions are associated with low efficiency and targeting abilities [13]. Therefore, LVX formulations have been improved through particle formulations with another component such as PLGA or PEG to increase lung targeting and overall efficiency [14].The second component of the hybrid NPs is decellularized extracellular matrix (dECM) that retains essential structural and functional components, such as collagen, laminins, elastins, fibronectins, and glycosaminoglycans, that can bring about healthy functional tissue after immune processes [15]. Previously, porcine lung ECM has been formulated into NPs (PL-ECM NPs) that possess pro-regenerative responses in macrophages and antimicrobial properties against common bacterial strains in vitro [16]. ECM also induces pro-regenerative phenotypes for productive remodeling in other native tissues [17,18]. Furthermore, as the CF ECM is dysregulated, the addition of healthy ECM proteins may induce pro-regenerative responses to the lung, bringing about the formation of healthy functional tissue shown in other native tissues [15,17,18,19]. 

The goal of combining these components is to reduce the amount of drug delivered to the lung by directly targeting infections by introducing additional antimicrobial agents, as well as attempting to address airway inflammation by harnessing the ECM’s regenerative properties. This study aimed to create novel hybrid LVX-ECM nanoparticles using electrospray deposition and characterize their effectiveness as a possible CF therapeutic. We set out to characterize the size and charge of the NPs to foreshadow potential mucus penetration capabilities. The particles were subjected to an in vitro dissolution assay to evaluate their release kinetics as well as a metabolic activity cell assay with human airway epithelial cells to determine the biocompatibility properties. Lastly, the NPs were cultured with common CF bacterial strains to evaluate any bacterial inhibition or antimicrobial effects.

## 2. Results

### 2.1. Nanoparticle Characterization

**Figure 1:**
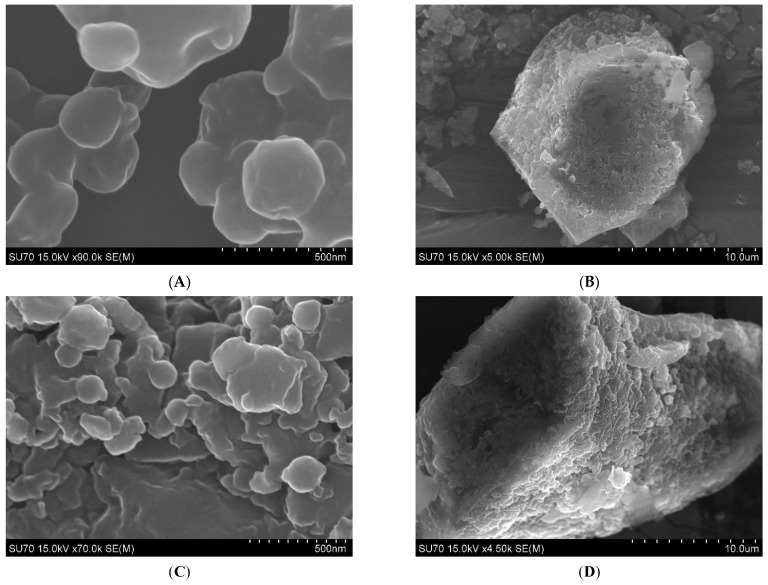
SEM images of 10:1 LVX-ECM NPs at the nanometer and micrometer scale (**A**,**B**) and 1:1 LVX-ECM NPs (**C**,**D**). NPs collected after being electrosprayed onto an aluminized steel sheet.

After electrospraying the nanoparticles, both the 10:1 and 1:1 LVX-ECM NPs show spherical and varying-sized particles at the nanoscale level (Figure 1A,C). The surface is smooth with no visible pores but some ridged and wrinkled points. At a lower magnification, both particle types show NPs embedded in a larger microparticle, suggesting a nano-into-micro (NiM) formulation (Figure 1B,D).

The ZetaSizer data shows the average size of the 10:1 LVX-ECM NPs to be ~525 nm and an average zeta potential of ~−6 mV, a neutral charge (Table 1). The 1:1 LVX-ECM NPs have an average size of ~300 nm and an average surface charge of ~−4 mV, within the targeted range. The PL-ECM NPs, created using the same electrospray parameters, were much larger than the LVX-ECM NPs, sizing at around ~2500 nm, with a zeta potential of ~−13 mV and a PDI of ~0.86. This indicates that electrospray parameters are highly sensitive to the loading material.

### 2.2. In Vitro Dissolution and High-Performance Liquid Chromatography

**Figure 2:**
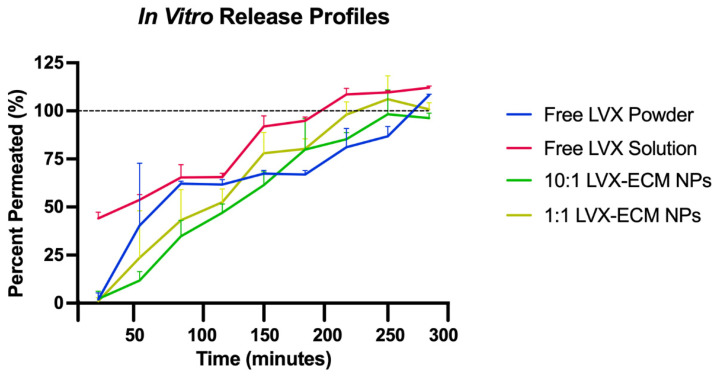
In vitro dissolution. Release profiles of 10:1 and 1:1 LVX-ECM NPs compared to equal amounts of free LVX. Data is displayed as mean ± SD (*n* = 3).

The in vitro drug release profiles were conducted using a Transwell system that mimics release in the lungs. The results show that free LVX as a solution is immediately released prior to addition to the Transwell and reaches 100% permeation the quickest (Figure 2). The free LVX powder, interestingly, shows a much slower release than the LVX solution, as well as the LVX-ECM NPs. The 10:1 and 1:1 LVX-ECM NPs show a similar profile and are not significantly different, but show a slowed release compared to the free LVX powder. All samples are completely permeated in between two and four hours.

### 2.3. MTT Assay

**Figure 3:**
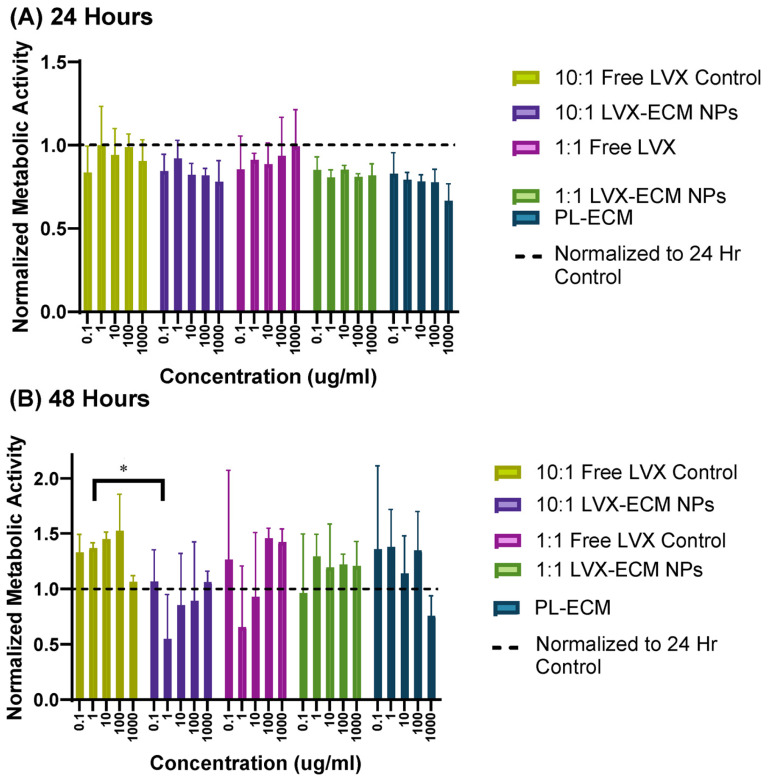
Calu-3 metabolic activity assay determined with MTT assay after 24 h (**A**) and 48 h (**B**) of incubation with LVX-ECM NPs and their corresponding controls. Data normalized to 24 h control to show metabolic changes between time points. Data is displayed as mean ± SD (*n* = 3) where * indicates *p* < 0.05.

The MTT results showed that after 24 h, all samples showed less metabolic activity than the media control, apart from the 10:1 free LVX control at the 1 µg/mL concentration, suggesting potential injury to the cells following addition of the NPs (Figure 3A). At the 48 h time point, all samples increased metabolic activity at least two concentrations, suggesting potential recovery and proliferative effects exhibited by the NPs (Figure 3B). The 10:1 free LVX control sample, at concentrations less than 1000 µg/mL, induced the highest metabolic activity. In contrast, the 1:1 free LVX control did not induce as much. Furthermore, the 1:1 LVX-ECM NPs had higher metabolic activity than the 10:1 LVX-ECM NPs, potentially showcasing that the increased amounts of PL-ECM within the NPs induce more proliferative effects. The PL-ECM NPs increased activity at all concentrations at 48 h except for the highest concentration.

### 2.4. Antimicrobial Assay

**Figure 4:**
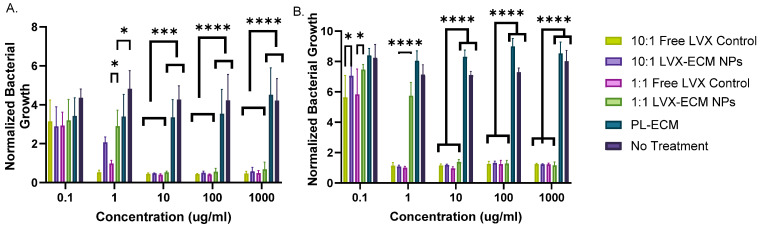
Bacterial inhibition assay measuring bacterial growth of *P. aeruginosa* (**A**) and *S. aureus* (**B**) when cultured with LVX-ECM NPs and their corresponding free LVX controls. Data is displayed as mean ± SD (*n* = 3) where **** indicates *p* < 0.0001, *** *p* < 0.0005 ** *p* < 0.001, and * indicates *p* < 0.05.

The results of the bacterial inhibition assay reveal similar outcomes for both bacterial strains. For *P. aeruginosa*, all samples inhibit bacterial growth at the 0.1 µg/mL concentration (Figure 4A). Concentrations from 0.1 to 1000 µg/mL exhibited antimicrobial properties, with the most inhibition starting from 10 µg/mL. The 10:1 free LVX control has consistent inhibition across concentrations from 1 to 1000 µg/mL, whereas the 1:1 free LVX control and the 10:1 and 1:1 LVX-ECM NPs inhibit around the same growth as 10:1 free LVX control at concentrations increasing from 10 µg/mL. At 1 µg/mL, the 10:1 and 1:1 LVX-ECM NPs inhibit less than the 10:1 free LVX control and 1:1 free LVX control, respectively. Similarly, for *S. aureus*, at concentrations higher than 1 µg/mL, all samples, except for the PL-ECM NPs, had similar bacterial inhibitions (Figure 4B). At 0.1 µg/mL, the 10:1 and 1:1 free LVX control had similar inhibition, whereas 1:1 LVX-ECM NPs and 10:1 LVX-ECM NPs had comparable inhibition. At 1 µg/mL, the 1:1 LVX-ECM NPs inhibited less than the free LVX and 10:1 hybrid NPs. Furthermore, at concentrations higher than 1 µg/mL, both free LVX and LVX-ECM NPs showed very similar bacterial inhibition. It is clear that 10:1 free LVX control displayed the most inhibition, which confirms that 10:1 LVX-ECM having more LVX than the 1:1 LVX-ECM NPs is more effective in bacterial growth inhibition. Interestingly, the PL-ECM NPs did not inhibit bacteria, potentially alluding to the addition of healthy ECM proteins aiding in bacterial growth.

## 3. Discussion

### 3.1. Nanoparticle Characterization

CF mucus mesh size imaged using atomic force microscopy (AFM) shows pore sizes of 160 to 1400 nm, and SEM images suggest 100 to 400 nm, suggesting the 1:1 LVX-ECM NPs could penetrate through the pores [7]. Additionally, particles of negative charges will encounter repulsive force with the mucus and may lead to aggregates on the airway lumen and be coughed out after inhaled administration [20]. Furthermore, neutral particles reduce the unwanted electrostatic interactions with DNA and mucins in the gel layer [20,21].

NiM particle formulations are another emerging method for more optimal storage and delivery of drugs to the lungs through DPI delivery [22] and were explicitly created to penetrate through CF mucus for bacterial or epithelial cell targeting. Additionally, as microparticles retain higher deposition rates within the bronchi between 1 and 6 um, the NiM formulation may be able to deposit within the bronchi, come into contact with the lung lining fluid, and break off the nanoparticles, which should then be able to attract and penetrate towards the mucus to target the infections. The collected aggregation of the particles may mean more optimal deposition to the mucus than the nanoparticles themselves; however, this should be tested in more depth with this specific NiM formulation.

The polydispersity index (PDI) of both NPs is relatively the same, indicating a broad particle size distribution (Table 1). Although most NP formulations are developed with an ideal size, a larger range of NP sizes may allow for further diffusion through CF mucus. CF mucus mesh sizes imaged using atomic force microscopy (AFM) show pore sizes of 160 to 1400 nm and SEM images suggest 100 to 400 nm [7]. To image the mucus, several techniques that dry and attach the mucus to the stage may alter the pore sizes and overall structure, leading to uncertainty in the measurements. Additionally, as the mucus pore sizes from each CF patient differ and are likely not all uniform within one patient or across several patients, the variance in size would allow bigger particles to become lodged in some pores while the smaller particles can continue to diffuse through the network and target the bacteria, targeting bacteria in the intraluminal mucus as well as bacteria attached to the epithelium. However, further testing of this diffusion could be measured through particle tracking analysis within patient mucus and additional mucus measuring research.

### 3.2. In Vitro Dissolution

The Transwell in vitro dissolution test results show that free LVX as a solution is immediately released prior to addition to the Transwell and reaches 100% permeation the quickest (Figure 2). The free LVX powder, interestingly, shows a much slower release than the LVX solution as well as both LVX-ECM NPs. The 10:1 and 1:1 LVX-ECM NPs show a similar profile and are not significantly different but show a slowed release compared to the free LVX powder. All samples are completely permeated between two and four hours. A poly (lactide-co-glycolide), or PLGA, LVX-loaded microsphere displayed a burst effect of 40% within the first 30 min and 70% between the two formulations, respectively [23]. Additionally, LVX-loaded chitosan microspheres displayed an immediate release of LVX before 30 min in all formulations when tested over a total of 3 h [23]. Another study with smart lipid NPs containing LVX showed a release of 80% and 60% of LVX within the first 4 h of the two formulations [24], which is comparable to the results from the LVX-ECM NPs in this current study, where LVX is permeated fully before 5 h. Based on past studies, the release of LVX is consistent and comparable, displaying expected results for the drug release profiles of free LVX and LVX-ECM NPs.

The Transwell in vitro dissolution test revealed that the 10:1 and 1:1 LVX-ECM NPs can slow the permeation and dissolution of LVX compared to free LVX solution but not the free LVX powder. As free LVX powder is around 1 micron in size, as shown in the previous ZetaSizer data, and the hybrid particles are nano-sized, the size does not affect the release of LVX. Logically, smaller-sized particles should be able to dissolve faster than larger particles, but this data shows that size of the sample is irrelevant. Therefore, the addition of ECM, regardless of size, can slow the release of the drug compared to free LVX. Contrarily, dry powder inhaler (DPI)-formulated microparticles of a much smaller size than untreated ciprofloxacin microparticles had a rapid dissolution [25]. However, in the same study, the presence of a mucolytic agent (Dornase alpha or N-acetylcysteine) with ciprofloxacin DPI particles reduced the dissolution rate compared to the ciprofloxacin DPI particles [25]. Therefore, additional studies with LVX NPs as suspensions can be compared to the LVX-ECM NPs to confirm the addition of ECM delays the dissolution of LVX.

### 3.3. Cell Viability Assay

The MTT assay had decreased levels in some ECM groups at 24 h. This may indicate that too-large amounts of added ECM components may potentially harm the cells. Additionally, size may play a role in compatibility, as the PL-ECM NPs were almost five times larger than the LVX-ECM NPs. To address this, PL-ECM NPs can be formulated with similar sizes as the hybrid NPs to discover the full effectiveness of the added ECM.

This data is consistent with other studies containing LVX NPs; for example, in culture with A549 lung epithelial cells, free LVX retained greater than 80% viability after 48 h [14]. The same research also confirmed that cationic particles induced a negative effect on cell viability, whereas anionic particles, like the LVX-ECM hybrid NPs, did not have a negative effect on viability [14]. This data agrees with our prior study on cell proliferation assays on A549 cells where PL-ECM NP concentrations less than 1000 µg/mL induced cell proliferation after 48 h of incubation [16]. However, more studies would need to be conducted to ensure that the proliferation of cells does not contribute to increased inflammation and aid in further lung damage. From our results, the LVX-ECM NPs show promising biocompatibility properties, as cells maintained or increased cellular metabolic activity. Additionally, this assay can be further improved by serum-starving the Calu-3 cells and testing on primary human epithelial tissue to ensure that any increase in overall metabolic activity is attributable to the LVX-ECM NPs and not media culture serum.

### 3.4. Antimicrobial Assay

Previously, PL-ECM NPs were tested against *Escherichia Coli*, *P. aeruginosa*, and *S. aureus* strains in nanogram concentrations [26]. The results showed that PL-ECM NPs could not inhibit E. coli growth but could elicit antibacterial activity against the other strains. Specifically, the PL-ECM NPs were able to slow the growth of *S. aureus* at lower concentrations; they inhibited *P. aeruginosa* growth at 6 h and slowed it over 24 h [26]. This suggests that at lower concentrations, PL-ECM NPs may be able to elicit antimicrobial properties rather than the high concentrations shown here.

An MIC for free LVX was concluded to be 0.625 mg/mL for isolated *P. aeruginosa* strains [23]. A previous study demonstrated that LVX concentrations of 0.5 and 1 mg/L enhanced *P. aeruginosa* bacterial killing; in addition, 2.5 and 4 mg/L enhanced *S. aureus* reduced cell survival over ciprofloxacin [27]. Based on the data in this experiment, the MIC is likely between 1 and 10 µg/mL, but this can be confirmed with further studies that include a smaller range of concentrations in between.

Decellularized ECM has previously been shown to induce antibacterial activity from different organs, including the lung [28,29,30]. PL-ECM NPs have been characterized to have an abundance of collagens compared to other ECM proteins, where the alpha 3 subunit of collagen VI has been shown to damage extracellular membranes and release cytoplasmic content of *Escherichia coli*, *S. aureus*, and *P. aeruginosa* [29]. However, it has also been shown that introducing ECM protein coatings with collagen IV advances microbial growth and biofilm formation [29]. This may suggest that adding ECM could act as an antimicrobial or facilitate the growth of bacteria, depending on the components.

There are potential limitations to our study. This assay could be improved by creating same-sized NPs of LVX and both hybrid LVX-ECM NPs to verify that size is not a factor in bacterial growth. Additionally, the assay could account for more concentrations and time points for a log phase graph for further determination of each component’s antibacterial activities. Further, the source material for our ECM was from the slaughterhouse. Future work would include ECM starting material from animals raised for clinical use and would likely require additional sterilization steps.

## 4. Materials and Methods

### 4.1. Materials

Levofloxacin anhydrous base and all analytical-grade HPLC solvents were purchased from Sigma-Aldrich, St. Louis, MO, USA. Innovative-grade US porcine lungs were supplied from Innovative Research, Novi, MI, USA. All other chemicals used were of pharmaceutical or analytical grade.

### 4.2. Decellularization of Porcine Lung

Previously, the ECMs from porcine lungs have been harvested and decellularized for therapeutic treatments in hydrogels and nanoparticles [31]. Briefly, sodium deoxycholate, DNase, and triton-X solutions were used as perfusive decellularizing detergents through the trachea and vasculature of the porcine lung. The resulting tissues were then extracted, lyophilized, and cryomilled into fine powders. The PL-ECM powder was stored at −80 °C.

### 4.3. Electrospray Deposition Process

To create the LVX-ECM nanoparticles, we used a previous methodology from a Heise lab protocol for an initial electrospray setup with different electrospray parameters [31]. A 10:1 and 1:1 ratio of LVX to ECM were used to create the LVX-ECM NPs. The LVX-ECM NPs and PL-ECM NPs were fabricated using the same electrospray parameters. Decellularized PL-ECM powder was dissolved in 80% *v*/*v* glacial acetic acid for 48 h at room temperature under gentle agitation. After the PL-ECM powder had been dissolved, LVX (Sigma-Aldrich, St. Louis, MO, USA) was then added to the digest with the addition of deionized water until the solvent reached 64% *v*/*v* glacial acetic acid. LVX was added in either a 10:1 or 1:1 LVX to ECM ratio for a concentration of 5 mg/mL of added powders, respectively. The resulting solution was then drawn up in a 10 mL syringe and a 26-gauge blunt tip needle was attached. Air was introduced to the system by sheathing the electrospray needle into a tube connected to an air supply to ensure complete drying of particles for collection. The syringe was placed in the syringe pump and set to a flow rate of 0.06 mL/h, a working distance of ~19 cm, and an applied voltage of −11 kV. The syringe pump was started and once the solvent in the syringe was emptied, the particles were collected and stored at room temperature.

### 4.4. Nanoparticle Size and Charge Characterization

The size and morphology of the LVX-ECM NPs were determined using scanning electron microscopy (SEM, Schottky Field Emission Gun; Hitachi High-Tech Corporation, Minato-ku, Tokyo, Japan). The NPs were attached to double-sided tape and mounted on the SEM stage; sputter coated with platinum; and run at different magnifications. The particles were also measured for size and zeta potential using a ZetaSizer (Malvern Instruments Ltd. ZetaSizer DTS0012, ZN90, Worcestershire, UK). The sample and nanoparticle charge, or zeta potential, was measured by placing the nanoparticles in a solution of 1 mL of sodium chloride.

### 4.5. In Vitro Dissolution and High-Performance Liquid Chromatography

To evaluate how the nanoparticles may be deposited in the lung, a protocol for an in vitro dissolution permeation profile was adapted to provide an in vivo relevant model for inhaled therapeutics [32]. As the LNPs would come into contact with lung fluid upon inhaled delivery, Simulated Lung Lining Fluid (SLLF) composed of magnesium chloride (0.095 g/L), sodium chloride (6.019 g/L), potassium chloride (0.298 g/L), sodium sulfate (0.071 g/L), calcium chloride (0.278 g/L), sodium acetate (0.574 g/L), sodium bicarbonate (2.604 g/L), sodium citrate (0.085 g/L), and sodium phosphate (0.123 g/L), dissolved in distilled deionized water, was formulated to mimic the lung environment (Sigma Aldrich, St. Louis, MO, USA [32]. Briefly, 2.6 mL of SLLF was placed into the receptor side of a 24 mm 6-well Transwell^®^ plate with a 0.4 μm Pore Polycarbonate Membrane Insert (Corning) [32]. Then, 1.5 mL of SLLF was added to free LVX, 10:1, and 1:1 LVX-ECM NPs (equivalent to 300 μg of LVX), respectively, and then added carefully on top of the polycarbonate membrane insert on the donor side. LVX powder was used as another control, where LVX (300 μg) was placed directly on the insert with the addition of SLLF on top. The 6-well plate was incubated at 37 °C with ~100% relative humidity. At different time points of 15, 30, 45, 60, 90, 120, 180, 240, and 300 min, starting when the NP suspension in SLLF was placed on the donor side, 200 μL of the receptor side fluid was taken for HPLC analysis and replaced with 200 μL of plain SLLF to retain the same volume.

The mobile phase used for HPLC analysis was composed of 400 mL of deionized water added to 1.23 g of tetrabutylammonium bromide (TBA), 75 mL of acetonitrile, 25 mL of methanol, and 1 mL of trifluoroacetic acid (TFA), which was then filtered and degassed. A C18 column was used for UV-HPLC analysis, reading at LVX detection of 295 nm (Waters 2995 Separations Module and Water 996 Photodiode Array Detector, Milford, MA, USA). The released LVX (%) was calculated as the experimentally measured amount of LVX as compared to the amount of LVX transferable to the receptor compartment (calculated based on the initial amount of LVX added to the Transwell donor compartment). The calculations are based on the initial amount of LVX added to the Transwell multiplied by 2.6/(2.6 + 1.5) based on the amounts added to the donor and receptor compartments; however, these volumes may have shifted during the experiment. This hinges on the idea that the theoretical dose of LVX would not release in only the receptor compartment but would rather be distributed across both the receptor and donor compartments. These reasons may explain the cumulative release exceeding 100% in the results.

### 4.6. MTT Assay

Human airway epithelial cells (Calu-3, ATCC, Manassas, VA, USA) were grown in Eagle’s Minimum Essential Medium (EMEM, Quality Biological, ThermoFisher, Grand Island, NY, USA) with L-glutamine, supplemented with 10% *v*/*v* fetal bovine serum (FBS, ThermoFisher), and incubated at an atmosphere of 5% CO2 at 37 °C. The cell culture media were replaced every 2 days.

Calu-3 cells were seeded at 10,000 cells per well on a 96-well plate (Falcon, Thermofisher, Grand Island, NY, USA) and incubated for 24 h. Media were then replaced with the free LVX powder equivalent to LVX present in LVX-ECM NP ratios, and the 10:1 and 1:1 LVX-ECM NP treatments were suspended in cell culture media at concentrations of 1000 μg/mL, 100 μg/mL, 10 μg/mL, 1 μg/mL, and 0.1 μg/mL with fresh media as the control. Once incubated for 24 h, the MTT labeling reagent was added at 10 μL per well and incubated for four hours. After incubation, 100 μL of solubilization buffer was added to each well and incubated overnight. The following morning, the absorbance was measured at 590 nm using the Agilent BioTek Epoch microplate spectrophotometer (Agilent Technologies, Santa Clara, CA, USA) to obtain the data. The data for each sample group has been normalized to the 24 h no treatment control of cells in media to show the changes in cell growth over time.

### 4.7. Antimicrobial Assay

An overnight culture of both Pseudomonas aeruginosa (PA01, donated from Dr. Hugh Smyth’s lab, University of Texas at Austin, Austin, TX, USA) and Staphylococcus aureus (MRSA, donated from Dr. Kimberly Jefferson, VCU, Richmond, VA, USA) was prepared by inoculating a colony in LB broth under shaking conditions. The culture was incubated at 37 °C at 200 rpm. The following day, 100 μL of bacterial culture was added to 10 mL of Luria Bertani broth (LB, Sigma Aldrich). The bacteria were read at an OD of 600 nm (Biomate 3, ThermoFisher) where the values were 0.6625 for *P. aeruginosa* and 1.2075 for *S. aureus*. After brief shaking and mixing of the culture in broth, 100 μL of the bacteria culture was plated with 100 μL of free LVX (equivalent to the amount present in the 10:1 and 1:1 NP formulations) and the 10:1 and 1:1 LVX-ECM NPs at concentrations of 1000, 100, 10, 1, and 0.1 μg/mL. A control of LB broth and bacteria with LB broth with no treatment were used as controls. After 24 h of incubation at 37 °C, each 96-well plate was read at 600 nm (Agilent BioTek Epoch microplate spectrophotometer, Santa Clara, CA, USA).

### 4.8. Statistical Analysis

All analyses were performed with GraphPad Prism 10 software. Statistical analysis of the in vitro dissolution assay, cellular metabolic activity assay, and antibacterial activity assay were performed by a two-way ANOVA with row and column-wise Tukey Tests of 99% confidence with an *n* = 3. Statistical significance was considered with a *p*-value of <0.05.

## 5. Conclusions

We have developed hybrid LVX-ECM nanoparticles as a potential therapeutic for airway bacterial infections. The fabrication and electrospray process created specifically sized and charged particles that may enable their deposition to common CF infections within the airway lumen and attachment to the airway epithelium. Compared to free LVX, the LVX-ECM NPs slowed drug dissolution and permeation regardless of size, showcasing the possibility that the ECM is able to enhance LVX release properties over time, which may be more effective as a treatment. The hybrid NPs were also non-toxic to airway epithelial cells and increased proliferation. When cultured with common CF strains, the NPs could inhibit the growth of bacteria. The combined properties of the LVX-ECM NPs prove they may be highly successful as a potential pulmonary infection therapeutic.

## Figures and Tables

**Table 1:** ZetaSizer data for the size, polydispersity index, and zeta potential of the LVX-ECM NPs after collection.

Sample	Size (nm)	Polydispersity Index (PDI)	Zeta Potential (mV)
10:1 LVX-ECM NP	525.9 ± 61.99	0.51 ± 0.01	−6.39 ± 1.03
1:1 LVX-ECM NP	298.7 ± 16.54	0.48 ± 0.06	−4.14 ± 0.44

## Data Availability

Data will be made available upon request.
